# Recall of Affective Responses to Exercise: Examining the Influence of Intensity and Time

**DOI:** 10.3389/fspor.2020.573525

**Published:** 2020-11-12

**Authors:** Malgorzata Maria Slawinska, Paul Anthony Davis

**Affiliations:** ^1^Department of Social Sciences, Institute of Sport–National Research Institute, Warsaw, Poland; ^2^Department of Psychology, Umeå University, Umeå, Sweden

**Keywords:** affective responses, affective valence, recall, dual mode model, exercise intensity

## Abstract

Affective responses to exercise are noted to be dynamic and frequently vacillate between positive and negative valence during physical activity. Recalled affect following exercise can influence anticipated affective responses to exercise and guide future behaviors. Research examining affective memory processes indicates that the recall of an experience can substantially differ from the actual experience and change over time. Grounded in the dual mode model (Ekkekakis, 2003), this study examines individuals' recall of exercise-related affect over a period of 2 weeks. Forty-two adults (26 women, 16 men; *M*_age_ = 29.64, *SD* = 5.69) completed two 20-min treadmill exercise trials in a randomized control crossover design; the trials were set either at a low or high exercise intensity based upon individuals' ventilatory threshold. Data analyses indicate that the affective responses to the low-intensity condition were evaluated more positively than in the high-intensity condition. Recalled affect fluctuated over a 2-week time period following both the low- and high-intensity exercise trials. A significant reduction at the 24-h recall measurement point was observed in both exercise intensity conditions. Implications for future research and health promotion interventions aiming to optimize affective responses to exercise are presented.

## Introduction

In attempts to increase understanding of the mechanisms underlying the global issue of physical inactivity (Troiano et al., 2008; Ding et al., 2016), contemporary research increasingly focuses on factors that influence individuals' perceptual experience of exercise (e.g., Kwan and Bryan, 2010; Ekkekakis et al., 2013; Hutchinson et al., 2020). Numerous studies highlight that positive affect experienced during exercise is a significant predictor of future exercise behavior (for review, see Rhodes and Kates, 2015); conversely, experiencing discomfort, unpleasant affect, or pain during exercise is likely to contribute to a discontinuation of engagement in physical activity (Ekkekakis, 2003; Brand and Ekkekakis, 2017).

Extensive research of affective responses to exercise has been guided by Russell's (1980) circumplex model of affect comprising the dimensions of valence and arousal; primarily studies have focused upon how pleasant versus unpleasant (i.e., valence) individuals perceive the experience of physical activity to be (e.g., Williams et al., 2012; Williams and Raynor, 2013). Over a period of 50 years of research, the widely promoted “feel good” effect of exercise has been scrutinized (Ekkekakis et al., 2011; Ekkekakis and Brand, 2019); further, popular claims extolling the virtues of high-intensity interval training related to the cliché “no pain, no gain” have been the focus of recent affect-related research (Stork et al., 2018; Niven et al., 2020; Roloff et al., 2020). Numerous studies highlight that affective states derived from exercise are not purely positive; rather, over the course of exercise, affective states vary, and typically shift between pleasant and unpleasant hedonic valence (Backhouse et al., 2007; Ekkekakis et al., 2008; Rose and Parfitt, 2012). One explanation forwarded in an attempt to outline the extent of variability in affective responses during exercise is the dual mode model (DMM; Ekkekakis, 2003; Ekkekakis and Acevedo, 2006). The DMM posits that, when exercise intensity is below the point of the ventilation threshold (VT), individuals tend to derive greater pleasure from exercise. However, when exercise intensity is high and exceeds an individual's VT, the experience tends to generate less pleasant affect and increase unpleasant affect. The shift in affective responses has been accounted for by the domination of interoceptive cues (e.g., pain in leg muscles or the perception of being out of breath), which are strongly associated with unpleasant affect (Ekkekakis et al., 2004, 2008).

Early studies of affective responses associated with exercise typically measured affect pre and post sessions with more recent research advancement incorporating measurement during activity (Backhouse et al., 2007; Ekkekakis and Brand, 2019). Further, it was presumed that, upon exercise completion, the evaluation and memory of affective responses associated with the task remained constant over time and reflected a composite affective state (e.g., Williams et al., 2008). However, research on behavioral economics and social cognition of the memory of affective experience (e.g., Kahneman et al., 1993; Baumeister et al., 2001; Miron-Shatz et al., 2009) highlight that, rather than a composite affective state, individuals tend to remember particular segments of an experience, and the recall is prone to changes over time. In particular, the memory of the affective experience, rather than the actual affective experience, plays a central role in individuals' decisions about whether or not the behavior is repeated (Kahneman et al., 1997; Fredrickson, 2000).

In light of the role of memory in exercise-related affect, more recent research has investigated how individuals perceive their affective responses during physical activity as well as how the affective experience is remembered and recalled over time (e.g., Zenko et al., 2016). In particular, individuals who complete physical activity with greater positive affect and a lower exercise intensity remember the experience of the entire exercise session as more pleasant in comparison with individuals who complete their exercise at a higher intensity (Hargreaves and Stych, 2013). In explaining this predisposition, it is suggested that individuals do not consider the entire duration of exercise experience; alternatively, salient pieces of information (i.e., the peak and end) form the composite memory of the experience (Fredrickson, 2000).

The remembered affective experience of physical activity has been noted to influence the anticipated affective responses to subsequent sessions of physical activity (Kwan et al., 2017; Davis and Stenling, 2020). Although this observation is important for physical activity promotion, a limitation that arises from the aforementioned studies relates to the timing of the post-exercise measurement of remembered affective experience. Specifically, Kwan et al. (2017) study collected remembered affect only once at a time point 5 min post-exercise; Davis and Stenling (2020) measured remembered affect twice, but at intervals that were in close proximity to the completion of physical activity (i.e., 90 s and 3 min) and commencement of the subsequent session (i.e., 10 min). Repeated measures of remembered affective responses to exercise collected over a longer period of time (e.g., the following day) may elucidate the dynamic changes in remembered affective states over time.

Recent studies examining the role of affect in physical activity and lifestyle behaviors have used ecological momentary assessments (EMA) to capture experiences in naturalistic settings and to better understand patterns of change that occur over time (e.g., Ivarsson et al., 2020; Smith et al., 2020). In particular, a review of studies using EMA determined that higher levels of positive affect predicted greater physical activity within the next few hours and vice versa; however, the negative affect–physical activity relationship was mixed (Liao et al., 2015).

A potential explanation for the mixed findings of negative affect may relate to the timing of measurements of affective states and physical activity; current affect is predominantly the focus of assessment and, in turn, is associated with subsequent activity (Kim et al., 2020). Recent research examining the recall of past affective experiences using EMA notes a recall bias (i.e., the tendency to overestimate and/or underestimate positive or negative past emotional experiences; Colombo et al., 2020); however, research in the domain of exercise has not examined temporal aspects of recalled affective responses to exercise using EMA.

Taken collectively, previous research suggests the examination of changes in exercise-related affect over time is likely to offer insight into how individuals recall the experience of exercise and potentially explicate mechanisms underpinning individuals' exercise behaviors (Backhouse et al., 2007). The present study aims to advance understanding of the links between affect and exercise by investigating individuals' recall of their affective experience associated with exercise. Guided by the DMM (based on individuals' VT), the present study examines individuals' affective experiences during two exercise trials (high vs. low intensity) as well as their recall of exercise related affect over a 14-day period following each trial. In line with previous research, we hypothesized that affect experienced during low exercise intensity would be more pleasant than during high exercise intensity. Further, we hypothesized that the memory of exercise-related affect would not mirror the affective experience during exercise and would fluctuate over the 14-day recall period in relation to the exercise intensity conditions.

## Methods

### Participants

The study sample consisted of 42 adults (26 women, 16 men; *M*_age_ = 29.64, *SD* = 5.69); at the time of recruitment to the study, participants completed a health survey to confirm that they did not suffer from any physical injury, had no history of cardiovascular, or respiratory disease, and were currently physically active and able to engage in moderate exercise. Participants were recruited via a generic email, which was sent to undergraduate and postgraduate students at the university where the data were collected. Upon agreement to take part in the study, participants provided written informed consent. The study received institutional ethical approval from the university where the data were collected.

### Measures

#### Affective Responses

The core affective dimension of valence was assessed by the Feeling Scale (FS; Hardy and Rejeski, 1989). The scale entails an 11-point bipolar measurement scale of pleasure–displeasure, ranging from +5 (*I feel very good*) to −5 (*I feel very bad*) with anchors at zero (*neutral*), participants were asked to rate “*How do you feel?”* Concurrent validity data have been previously reported by Hardy and Rejeski. The FS was used to measure affective responses prior to, during, and after each of the exercise trials (i.e., low and high exercise condition). In consideration of the nature of the experiment, a single-item rating was evaluated as most adequate as it limited the burden of data collection being placed upon participants during exercise bouts.

#### Remembered Pleasure

Recall of affective experience was measured with the Global Affective Evaluation Scale (GAE; Schreiber and Kahneman, 2000), which has previously been used to measure the overall amount of pleasantness or unpleasantness experienced during exercise (Hargreaves and Stych, 2013). The scale has a range from −10 (*very unpleasant experience*) through 0 (*neutral experience*) to +10 (*very pleasant experience*); participants were asked to “*Please rate the overall amount of pleasantness of unpleasantness that was experienced during the previous exercise trial*.”

The Rating of Perceived Exertion Scale (Borg, 1998) was used to assess whole-body ratings of perceived exertion (RPE). Participants stated the number that reflected how difficult the exercise felt on a 6–20 scale, ranging from 6 (*no exertion at all*) through 13 (*somewhat hard*) to 20 (*maximal exertion*). In line with recommendations from Borg (1998), participants were provided standardized instruction on how to use the scale and time to practice during the familiarization session.

### Procedure

#### Maximal Graded Treadmill Test

In order to determine individuals' ventilation threshold (VT), prior to the first exercise trial, maximal aerobic capacity (VO_2max_) data were collected. Participants competed the incremental treadmill test based on the Balke-Ware test protocol (ACSM, 2006). The analysis of pulmonary gas exchange was recorded continuously with an online gas analyzer (Oxycon Pro, Jaeger, Germany). The attainment of maximal aerobic capacity was verified by at least two of the following criteria: (i) a peak or plateau in oxygen consumption (changes <2 ml. kg^−1^ min^−1^) with increasing workload; (ii) reaching age-predicted maximal heart rate (i.e., 220—age); and/or (iii) a respiratory exchange ratio of at least 1.1. V_O2peak_ was established at the point where V_O2_ attained the highest value after reaching the criteria. The V-slope method was then used to determine individuals' VT. V_CO2_ was plotted against V_O2_, and from visual examination of the graph, VT was determined at the point where the first inconsistent increase in V_CO2_ occurred (Gaskill et al., 2001).

#### Exercise Sessions

Upon arrival at the laboratory, participants were asked to complete a self-reported measure of affective responses (FS). Individuals were randomly assigned (using a web-based online randomizer application; random.org) to one of the two exercise intensity conditions: 10% above-VT and 20% below-VT (based on previously determined individual point of VT). Depending on the randomization, participants were asked to run for 20 consecutive minutes on a treadmill that was set at either a high (above-VT) or low (below-VT) pace. Preliminary baseline measurements were collected immediately prior to commencing the exercise trial; specifically, affective responses (FS) were recorded immediately before the task commenced and repeatedly every 5 min for the remaining 20 min of the exercise task along with the measure of RPE (Borg 10). For the purpose of capturing fluctuation in affective responses over time, the measure of FS was recorded 1 min into a cool-down period of light running, as well as 5, 10, and 15 min post exercise, followed by another 4 points in time (i.e., 4, 24 h, 7, and 14 days post-exercise at the same time of day as when the exercise trial was completed). Participants were asked to complete the measure of GAE at 5 and 15 min post-exercise, and then at another 4 points in time (i.e., 4, 24 h, 7, and 14 days). Participants were asked to attentively follow the instructions for the post-exercise measurement by reporting their affective evaluation in a timely fashion via an online survey (surveymonkey.com). The responses were prompted by a notification sent to participants' mobile phones via text message approximately 30 min before the designated time of the measurement.

Following a 2-week period of time from the first exercise trial, the second exercise trial was undertaken. The second trial was completed using the same measurement protocol as detailed in the first trial. In the second exercise trial, those participants who previously exercised at an above-VT pace now performed the below-VT condition, whereas those who performed the first trial in the below-VT condition exercised at an above-VT pace in the second trial. To reduce expectancy effects, the participants were informed that the intensity may range from a low to a high exercise intensity based on their individual capacity measured at V_02max_ test taken previously.

### Statistical Analysis

To examine within-subject changes in current affect (FS) and recalled affective experience (GAE) over time at a group level, repeated-measure ANOVAs were used. The EMA method was used to analyze within-subject variations in current and recalled affect in everyday life. EMA has been successfully used in previous studies examining mood and physical activity (Hausenblas et al., 2008; Kanning and Schlicht, 2010). All statistical procedures were conducted in IBM SPSS version 20 statistical software.

## Results

Data collected from post-exercise measurement points showed that a number of participants failed to complete all of the post-exercise survey questions over the course of 28 days (*N* = 13; 32.5%). However, IBM SPSS 20 does not account for missing data from participants who omit one or more measurement points over the 2-week periods of data collection. Subsequently, data from 27 participants were subject to a maximum likelihood estimation using the expectation maximization algorithm to estimate and impute missing data based on the available responses. This method has previously been used in longitudinal studies in an exercise context (Helfer et al., 2015). In order to avoid estimating data based on inadequate information, participants were excluded if they were missing more than 40% of the follow-up surveys (van Ginkel and Kroonenberg, 2014). The data (GAE, FS) collected during the two experimental exercise conditions (below VT, above VT) were included in the estimation algorithm as predictors of missing values, and estimates were constructed by applying the Missing Value Analysis function.

### Affective Responses

**Figure 1:**
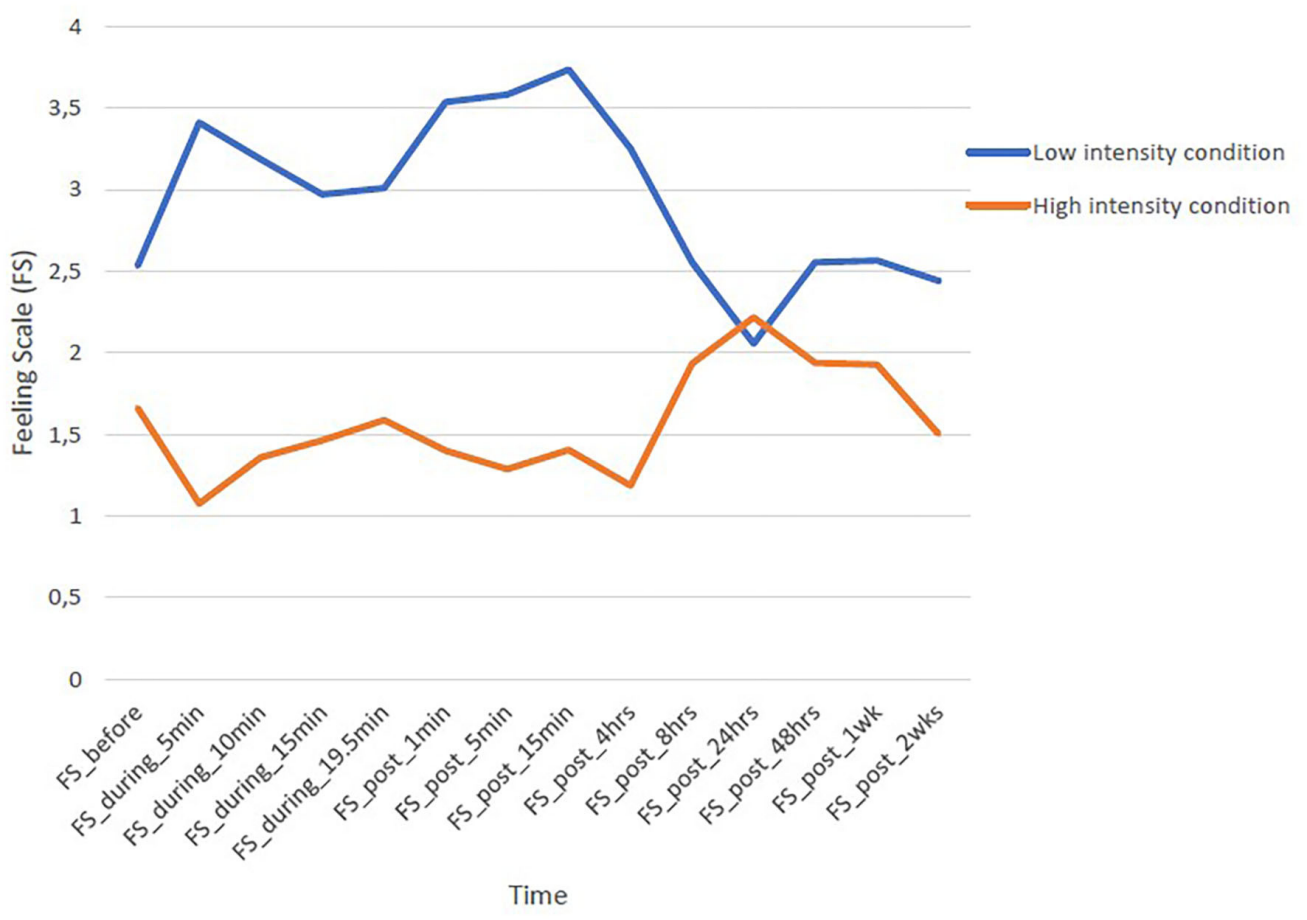
Changes in feelings states (FS) prior, during and after low and high exercise intensity.

Repeated-measures ANOVAs showed significant changes in affective responses over time during and after low-intensity exercise *F*(13, 38) = 6.85, *p* < 0.001, η^2^ = 0.15 and high-intensity exercise *F*(13, 38) = 7.49, *p* < 0.001, η^2^ = 0.16. Figure 1 illustrates patterns of how individuals feel with changes prior, during, and after the low and high exercise intensity.

### Recall of Affective Experience

The descriptive data reflecting the recall of affective experience is displayed in Table 1.

**Table 1:** Descriptive statistics of recall of affective experience (GAE).

**Time**	**1 min post**	**5 min post**	**15 min post**	**4 h post**	**8 h post**	**24 h post**	**48 h post**	**1 week post**	**2 weeks post**
Low intensity *M* (*SD*)	6 (3.01)	5.69 (3.30)	6.28 (3.11)	6.30 (2.86)	6.29 (3.16)	5.10^**^ (3.79)	5.58 (4.06)	6.44 (3.20)	5.77 (3.14)
High intensity *M* (*SD*)	3.1 (4.37)	3.56 (3.39)	2.82 (5.10)	3.07 (4.97)	2.71 (5.16)	1.97^*^ (5.04)	2.64 (5.18)	2.77 (4.64)	3.54 (4.98)

** *p < 0.01*,

* *p < 0.05*.

Results show the recalled exercise-induced affective valence that was reported in the postexercise evaluations was subject to fluctuations. The repeated-measure ANOVAs indicate significant changes in recalled affect in the low-intensity exercise trial *F*(8, 39) = 3.14, *p* = 0.012, η^2^ = 0.29, and in the high-intensity exercise trial *F*(8, 39) = 3.89, *p* = 0.03, η^2^ = 0.34. Further, pairwise analyses revealed significant differences between the 8 and 24 h post-exercise measurement points in the low-intensity condition *t*(39) = 3.22, *p* < 0.01, *d* = 0.75 and high-intensity condition *t*(39) = 2.02, *p* < 0.05, *d* = 0.55. Overall, the low-intensity condition was evaluated more positively than the high-intensity condition.

### Predicting Recalled Affective States at 24 h Post-Exercise

Multiple regression analyses were used to examine the impact of affect experienced during the exercise trials and affect remembered from exercise on the 24-h measurement point of recalled affective state. In the low exercise intensity condition, four measurement points of feeling states obtained during exercise together with the first five recall measurements of affective states, measured with GAE at 1, 5, 40 min, 4, and 8 h accounted for 88% of variance of the recalled affective state at the 24-h measurement point *F*(9, 36) = 21.89, *p* < 0.001. Coefficient analysis shows that two independent variables, recall at 4 and 8 h were significant. In the high exercise intensity condition, the four measurement points of feelings states obtained during exercise together with the first five recall measurements of affective states explained 92% of the variance of the 24 h measurement point *F*(9, 36) = 21.89, *p* < 0.001) with an *R*^2^ = 0.92.

## Discussion

The aim of the present study was to examine how individuals recall their affective experience of two exercise conditions (i.e., low and high intensity). The results indicate that the recall of affective experiences after exercise continues to fluctuate regardless of whether the exercise intensity is low or high. Therefore, these findings contrast with the commonly held presumption that post-exercise affect remains constant after exercise is completed. However, these results are in line with research suggesting that the affective memory of exercise is comprised of information that does not reflect the exact affective experience during exercise (Hargreaves and Stych, 2013). The results of the present study indicate that high-intensity exercise is evaluated less positively than low intensity exercise. These finding are in line with the DMM, which explains that, during the low-intensity exercise, there is a larger role of cognition, and the exercise is typically perceived as more pleasant, and during high-intensity exercise, a heavier role is played by physical factors, such as interoceptive cues (e.g., pain in the leg muscles or perception of being out of breath), which are strongly associated with negative unpleasant affect (Ekkekakis, 2003; Ekkekakis et al., 2004, 2008). The current study findings can also be extended to related research linking the experience of exercise with adherence; specifically, exercising at high intensity is associated with less positive experience and significantly lower rates of adherence than low or moderate exercise intensity (DaSilva et al., 2011).

A key finding arising from the present study notes that the recall of affective responses declines in reported valence significantly at the 24-h post-exercise measurement point. The research literature offers two possible explanations for a decrease in the recall of affective responses at 24 h post-exercise, one is a termination of the anxiolytic effect from exercise (Ebbesen et al., 1992; Salmon, 2001; Kwan and Bryan, 2010). A study by Ebbesen et al. (1992) highlights that exercise attenuates perceived stress for a period between 1 and 3 h after exercise, but the effect does not extend to 24 h. Kwan and Bryan (2010) demonstrate that, after a submaximal bout of exercise, an individual's level of tranquility is raised above baseline levels with measurements of tranquility recorded at 15 and 30 min post-exercise; the observed effect was maintained across the two time points. The authors conclude that, if increased exercise-related feelings of tranquility persist throughout the day, regular exercise behavior may reduce stress.

Another plausible explanation for the observed decline in affective valence 24 h post-exercise relates to delay onset muscle soreness (DOMS; Cheung et al., 2003) following unaccustomed exertion, which tends to occur ~4 to 8 h after exercise. Participants' recall of affect at the 24-h measurement point could have been biased by the salience of DOMS when evaluating the exercise experience. These explanations are two potential mechanisms underlying the affective decline observed in recall at 24 h; however, further research is required to test these explanations.

Some studies show that long-term exercise is positively associated with positive affect during and after exercise (Arent et al., 2000; Schneider et al., 2009); affect can provide feedback regarding the exercise experience and serve as a powerful motivational tool in exercise adherence. Affective feedback can promote approach or avoidance behaviors, either toward or away from future repeat experiences, depending on how the experience is remembered and the outcomes that are anticipated (Baumeister et al., 2007; Brand and Ekkekakis, 2017). In regards to exercise, it is common that, upon completion, one feels positive and may likely think of engaging in exercise in the following days. However, contrary to immediate post-task planning, the actual decision to initiate another bout of exercise occurs at a later point in time. The current study shows that the memory of exercise-induced affect is prone to changes over time. At a time of decision making, the recall of affective experience from exercise is likely to be an important factor to construct the anticipatory affective states. Previous study shows that individuals who anticipate a more pleasant affective experience from exercise report higher, positive affective responses during the subsequent exercise trials (Davis and Stenling, 2020). Findings obtained from the present study raise important applied implications for professionals aiming to optimize health and high performance as well as the development of training programs designed to maximize exercise adherence. Training plans should consider temporal fluctuations in the recall of exercise-related affect and implement strategies that attempt to minimize observed patterns of diminished positive affect.

Prospective studies should further examine the process underlying individuals' decision to engage in repeat exercise by attempting to understand how recalled exercise-induced affect interacts with evaluative processes that guide exercise behavior. Similarly, future studies may consider using qualitative research methods and to examine the recall of exercise-induced affect 24 h postexercise. Further study is also warranted to determine if the observed drop in positive recalled affect at 24 h postexercise is present if individuals are participating in a longer program of exercise and are scheduled to exercise again within the following days.

### Limitations and Future Directions

A clear limitation of the current study is that, during the 2-week recall period, neither meaningful life events nor an actual level of physical activity were controlled for, which could have an impact upon the recollection of the previous affective experience of the exercise activity. Although participants were strongly advised that they should refrain from physical activity, it cannot be presumed with certainty that they adhered to the advice. Furthermore, the potential explanations that were offered to account for the decline in recall of affective experience (i.e., the termination of anxiolytic effect of exercise and DOMS) are only hypothetical; individuals who took part in the study were not specifically examined for these potential mechanisms. Henceforth, future studies should undertake the examination of these proposed explanations and assess whether individuals actually experience a termination of anxiolytic effect or a DOMS during post-exercise evaluation.

Based on the findings from the study, it is important for prospective studies to further examine when exactly people make decisions to engage in exercise in order to understand how exercise-induced affect that is recalled over time interacts with evaluative processes and as a consequence how it is integrated into decision making guiding future exercise behavior. Similarly, future studies may consider using a qualitative approach and more in-depth inquiry to further examine recalled affective experience from exercise with a particular focus on the 24-h post-exercise time point. Prospective studies may also examine if the 24-h post-exercise drop in the recall of affective experience persists if individuals are participating in a longer program of exercise and have scheduled another exercise session within the following days.

### Conclusion

In light of the escalating global issue of physical inactivity, the results from this study highlight an important underlying factor of exercise behavior by delineating how exercise-related affective states are remembered over time. Individuals' prospective exercise participation is guided by the recall of affective states experienced during the exercise. In a context where many competing activities can inhibit exercise, targeting post-exercise affective evaluation may enhance the effectiveness of physical activity interventions.

## Data Availability Statement

The raw data supporting the conclusions of this article will be made available by the authors, without undue reservation.

## Ethics Statement

The studies involving human participants were reviewed and approved by University Research Ethics Committee (REC) at Northumbria University. The patients/participants provided their written informed consent to participate in this study.

## Author Contributions

MS planned and designed the study. MS and PD contributed to the design and implementation of the research, to the analysis of the results and to the writing of the manuscript.

## Conflict of Interest

The authors declare that the research was conducted in the absence of any commercial or financial relationships that could be construed as a potential conflict of interest.
